# Who Is Exposed to Secondhand Smoke? Self-Reported and Serum Cotinine Measured Exposure in the U.S., 1999–2006

**DOI:** 10.3390/ijerph6051633

**Published:** 2009-05-14

**Authors:** Wendy Max, Hai-Yen Sung, Yanling Shi

**Affiliations:** Institute for Health & Aging, University of California, San Francisco, 3333 California Street, Suite 340, San Francisco, CA 94118, USA; E-Mails: hai-yen.sung@ucsf.edu (H.Y.S.); yanling.shi@ucsf.edu (Y.S.)

**Keywords:** environmental tobacco smoke, secondhand smoke (SHS), passive smoking, serum cotinine, self-reported exposure

## Abstract

This study presents self-reported and serum cotinine measures of exposure to secondhand smoke (SHS) for nonsmoking children, adolescents, and adults. Estimates are disaggregated by time periods and sociodemographic characteristics based on analyses of the 1999–2006 National Health and Nutrition Examination Survey. Self-reported exposure rates are found to be highest for children, followed by adolescents and adults. Important differences in exposure are found by socioeconomic characteristics. Using serum cotinine to measure exposure yields much higher prevalence rates than self-reports. Rates of SHS exposure remain high, but cotinine levels are declining for most groups.

## 1. Introduction

Secondhand smoke (SHS) exposure, also known as passive smoking or exposure to environmental tobacco smoke, is known to be associated with a number of health effects, including respiratory illness, cancer and heart disease. Exposure has been shown to have an impact on children, adolescents and adults. These negative health impacts have been recently documented in several substantial reports which summarize the literature linking SHS exposure to respiratory effects in children and adult effects on cancer, cardiovascular disease and respiratory disease [1,2]. More specifically, SHS exposure has been shown to cause lung cancer [3], respiratory disease, particularly in children [4,5], and heart disease [6–8]. Recent evidence adds breast cancer in young women to the list of SHS related diseases [2].

Three recent studies have used the National Health and Nutrition Examination Survey (NHANES) data to estimate SHS exposure in the U.S. Pirkle and his colleagues [9] analyzed the NHANES III data from 1988–1991 and showed that serum cotinine levels indicate broader exposure to SHS than self-reported data. They found that the highest cotinine levels were for children, non-Hispanic Blacks, and adult males. In a later study, Pirkle and colleagues looked at trends in SHS exposure from 1988–2002 using serum cotinine concentration, and found that there have been substantial declines in exposure over this time period, though children and non-Hispanic Blacks have higher levels of exposure than others [10]. Schober, Zhang, and Brody [11] compared SHS exposure between 1988–94 and 1999–2004. They found that the percentage of nonsmokers with self-reported home exposure and the percentage with detectable serum cotinine declined between the two time periods. The rate of decline was smallest for children followed by adolescents. An analysis of the National Health Interview Survey found that SHS exposure at home, measured as the number of days per week someone smoked in the home, declined more substantially between 1992 and 2000 than would be predicted by the decline in active smoking [12]. Using data from the Medical Expenditures Panel Survey (MEPS), Machlin, Hill, and Liang [13] report that children who were older, poorer, and lived with adults with less education, were most likely to be exposed to SHS at home, as indicated by their living with at least one adult smoker. They do not report whether or not the person actually smoked inside the home.

Studies that compare the rates of exposure to SHS among racial/ethnic groups have generally found that among nonsmokers, African Americans have the highest rates of exposure. This has been reported for young adults using both cotinine concentration and self-reported number of hours of exposed per week [14], for women using self-reported number of days exposed per week [15], and for children using urinary cotinine levels and self-reported number of hours exposed [16]. However, it must be noted that when socioeconomic indicators were taken into consideration in one study, the higher rate of exposure among African American women was no longer found [15]. Hispanics have been found to have relatively low exposure rates, using both cotinine concentration and self-reported number of hours exposed [16]. Among nonsmoking Hispanic women of reproductive age, exposure to SHS differed by country of origin, according to an analysis of the Hispanic Health and Nutrition Examination Survey [17]. Exposure at home, indicated by whether or not someone in the household smoked, was found to be 31–62% for Mexican-American women, 22–59% for Puerto Ricans, and 40–53% for Cuban-Americans during 1982–1984 in the U.S., depending on age. For all groups, the youngest women (age 12–19) had the greatest exposure. For Mexican-Americans, exposure decreased with age, but for Puerto Ricans and Cuban-Americans, the pattern was less clear. At work, exposure was indicated by whether or not the respondent worked near someone who smoked in their presence. Work exposure was 22–35% for Mexican-Americans, 28–33% for Puerto Ricans, and 33–49% for Cuban Americans during the same period from 1982–1984. There was no pattern for work exposure by age. Pirkle and colleagues [9] reported that non-Hispanic Blacks had the highest exposure to SHS according to both serum cotinine measures and self-reported exposure. Findings from an analysis of the MEPS indicate that non-Hispanic white and black children were more likely than Hispanic children to live with at least one adult smoker [13].

Previous studies that have examined SHS exposure use several different measures of exposure, including serum cotinine concentration, urinary cotinine concentration, amount of time exposed, whether or not someone in the household smoked in the home, whether or not they lived with a smoker, and whether or not someone in the workplace smoked in their presence. The results are generally consistent, though subtle differences have been found depending on the measure used.

The goal of this study is to analyze the pattern of SHS exposure using three measures of SHS exposure: self-reported exposure, whether or not someone has a detectable serum cotinine level, and the actual level of serum cotinine. We will compare the patterns of exposure over time from 1999 to 2006 and by sociodemographic characteristics for different age groups. We will also compare the results from self-reported SHS exposure and cotinine measured exposure estimates. We extend the previous research by focusing on more recent years, and looking at how patterns of exposure differ in an era when all exposure rates are much reduced from previous levels.

## 2. Methods

### 2.1. Data Source

We analyzed the data from the 1999–2006 National Health and Nutrition Examination Survey (NHANES), which contains a nationally representative sample of noninstitutionalized civilian persons of all ages who were selected based on a complex sampling design. The NHANES is a household survey conducted by the National Center for Health Statistics of the Centers for Disease Control and Prevention to assess the health and nutritional status of adults and children in the U.S. Survey participants complete a home interview followed by a physical examination in a mobile examination center (MEC). The home interviews collect each participant’s demographic and socioeconomic characteristics, dietary patterns, health-related conditions, smoking and tobacco use status, and exposure to SHS. The physical examinations consist of medical, dental, and physiological measurements, as well as laboratory tests including blood and urine samples. During examination in the MEC, blood samples are drawn for serum cotinine analysis for all individuals age 3 and older.

Since 1999, the NHANES has been conducted on a continuous basis with data released every two years. Thus, between 1999 and 2006, there were four data release cycles: 1999–2000, 2001–2002, 2003–2004, and 2005–2006. Also, beginning in 1999, non-Hispanic Blacks, Mexican Americans, adolescents 12–19 years, older adults (60+ years), and low-income persons have been oversampled to improve the reliability of the statistical estimates for these subgroups. The response rate of the 1999–2006 NHANES was 81.4% for the home interview and 77.9% for the MEC examination [18].

### 2.2. Study Sample

While smokers may also suffer ill effects from SHS exposure, it is challenging to separate the health impacts of active and passive smoking. Therefore, in this study we focused only on nonsmokers. Our study sample comprises three age groups of nonsmokers: children (age 3–11), adolescents (age 12–19), and adults (age 20 and older). We excluded anyone less than three years old because serum cotinine was not measured in this group. The age cutoffs for adolescents and adults were used to be consistent with two sets of NHANES questionnaires about cigarette smoking and tobacco use. As part of the home interview, respondents aged 20 and older were asked about their history of cigarette smoking and other tobacco use. These individuals (i.e., adults aged 20 and older) were defined as “current smokers” if they answered that they have smoked at least 100 cigarettes in their lifetime and currently smoke cigarettes everyday or some day, or that they have used other tobacco products including pipe, cigar, snuff, or chewing tobacco at least 20 times in their entire life and now use these tobacco products. As part of the audio computer-assisted self interview during the physical examination in the MEC, respondents aged 12 to 19 years, who answered that they have tried cigarette smoking, were asked how many days they smoked cigarettes in the past 30 days. These individuals (i.e., adolescents aged 12–19) were defined as “current smokers” if they answered that they had smoked cigarettes at least one day in the past 30 days. In addition to these two sets of questionnaires, all respondents aged 12 and older were also asked about their use of tobacco products (cigarettes, pipes, cigars, chewing tobacco, snuff, nicotine patches, nicotine gum, or any other product containing nicotine) in the past five days during the physical examination in the MEC.

We considered all children to be nonsmokers. For adolescents and adults, nonsmokers were those who were not “current smokers” as defined above, had not used any tobacco products in the past five days, and had serum cotinine levels ≤ 10.0 ng/mL. While the choice of an arbitrary serum cotinine cutoff may lead to classifying some light smokers as nonsmokers, or excluding some nonsmokers who are heavily exposed to SHS, others have reported that using a cutoff of 10 or 15 mg/mL has little impact on results [9,10]. For the analyses of self-reported SHS exposure, individuals with responses to home exposure questions of missing, “don’t know”, or “refused” were excluded. The final sample size for nonsmokers was 25,329 for 1999–2006. For the analyses of serum cotinine measured exposure, individuals with missing serum cotinine data were excluded; the final sample size for nonsmokers was 24,001 for 1999–2006.

### 2.3. Measures of SHS Exposure

We analyzed exposure to SHS using two types of measures: self-reported exposure and cotinine measured exposure. Self-reported exposure to SHS was defined on the basis of the following NHANES questions: for each household participating in the survey, one family member was selected to answer whether any of the household members smoked cigarettes, cigars, or pipes anywhere inside the home. If any household member smoked, then all members of that household were classified as being exposed to SHS in the home. Respondents aged 16 and older who reported that they had a job or business in last week were asked: “At this job or business, how many hours per day can you smell the smoke from other people’s cigarettes, cigars, and/or pipes?” Those who reported one hour or more were defined as being exposed to workplace SHS. For children and adolescents, we only examined their SHS exposure status in the home. For adults, we examined both exposure status in the home and exposure status at work.

Serum cotinine, a metabolite of nicotine, was assessed in the NHANES using an isotope dilution, liquid chromatography tandem mass spectrometry method [9]. Analyses were conducted at the Centers for Disease Control and Prevention laboratory at the National Centers for Environmental Health. In the 1999–2000 survey cycle, serum cotinine measurement was designed to detect levels as low as 0.05 ng/mL. Beginning in 2001–2002, a similar but more sensitive cotinine assay allowed for a lower detectable limit of 0.015 ng/mL. We defined two kinds of cotinine measured exposure: whether or not someone has a detectable serum cotinine level of 0.05 ng/mL or greater, and the actual level of serum cotinine. The detectable limit of 0.05 ng/mL was used so that our data would be comparable across all years.

### 2.4. Measurement of Sociodemographic Characteristics

We considered the following sociodemographic variables: gender, race/ethnicity (non-Hispanic White, non-Hispanic Black, Mexican-American, other Hispanic, other race/ethnicity), country of birth (U.S., Mexico, elsewhere), poverty status and educational level. Poverty status was measured by the poverty income ratio (PIR) developed by the U.S. Census Bureau [19]. PIR is the ratio of family income to the family’s appropriate poverty threshold taking family size into account [20]. We classified PIR into four categories: poor (0.0–0.99), low income (1.0–1.99), mid income (2.0–3.99), and high income (≥ 4.0). Values less than 1.0 indicate that family income is below the official poverty threshold. Education level was classified into less than high school, high school graduate or General Educational Development (GED) diploma, and more than high school graduate. The education level for children and adolescents was determined by the status of the reference person in their household.

### 2.5. Statistical Analysis

All analyses accounted for the complex survey design and incorporated sampling weights that adjusted for unequal probabilities of sample selection, nonresponse, and sample noncoverage. All the statistical analyses were carried out using SAS version 9.1 [21] PROC SURVEYMEANS, PROC SURVEYREG, and PROC SURVEYLOGISTIC procedures which adjust for complex sample design when variance estimates are computed. We calculated the percentages of nonsmokers with self-reported SHS exposure and the percentages of nonsmokers with detectable serum cotinine levels. We also calculated the geometric means of the serum cotinine levels among nonsmokers. Because the cotinine levels were not normally distributed, we log-transformed the actual values before performing any analyses [9,10]. For nonsmokers whose serum cotinine level was below the detectable limit, we used an estimated level of 0.035 ng/mL (ie, the detectable limit, 0.050 ng/mL, divided by the square root of 2) [9,22,23]. Exposure prevalence rates and geometric means as well as their 95% confidence intervals (CIs) were calculated by age group (children, adolescents, adults), time period (1999–2000, 2001–2002, 2003–2004, 2005–2006), and sociodemographic characteristics.

To assess whether there was a significant time trend in SHS exposure during the eight years from 1999 to 2006, we fit an ordinary least squares regression model for all nonsmokers within each age group. In the model, the dependent variable is one of the three SHS exposure measures and the independent variable is the “time trend” variable with the value of 1 for 1999–2000, 2 for 2001–2002, and so forth. If the coefficient of the time trend variable is negative and statistically significant, it indicates there was a decreasing trend. For the analyses of the relationship between sociodemographic characteristics and SHS exposure, the four waves of data were combined. Differences in self-reported exposure among population subgroups were evaluated using multivariate logistic regression models which adjust for gender, race/ethnicity, country of birth, education level, and poverty income ratio. Adjusted odds ratios and 95% confidence intervals (CIs) were estimated. Differences in geometric means of cotinine levels among sub-populations were evaluated using multivariate linear regression models of log-transformed cotinine levels by adjusting for sociodemographic covariates We considered estimates to be statistically significant if the two-sided P-value < 0.05.

## 3. Results

### 3.1. SHS Exposure over Time

Table 1 shows the prevalence of self-reported exposure, prevalence of detectable serum cotinine, and geometric mean of cotinine levels among nonsmokers for the four waves of data and the three age groups. Consistent with earlier studies, the serum cotinine measure indicates a much higher prevalence of SHS exposure than self-reports. For all the three age groups, the difference in exposure rates is nearly threefold. Over the four time periods from 1999 to 2006, children and adolescents were found to have self-reported home exposure rates in the range of 14.6–23.7% and 15.2–23.4% respectively, compared to cotinine measured exposure rates of 50.9–65.2% and 47.0–63.2% respectively. Adult total self-reported exposure includes both exposure status in the home and exposure status at work. Work only exposure rates exceeded home only exposure rates. Very few people, 1.1 percent or fewer of the adult nonsmokers, were exposed in both settings. During the three time periods from 1999 to 2004 for which work and home exposure data are available, adults’ total self-reported rates were in the range of 13.1–15.7% compared to cotinine measured exposure rates of 37.3–47.4%. For both self-reported and cotinine measured prevalence over all periods, children had the highest SHS exposure rates. Adolescents’ rates were a bit lower except for the self-reported rates during 1999–2000 and 2005–2006, and adults had the lowest rates. The geometric mean of cotinine levels for children ranged from 0.11 to 0.18 ng/mL during the 8-year period. For adolescents the range was 0.09 to 0.14 ng/mL and for adults the range was 0.06 to 0.08 ng/mL. During every time period, children had the highest mean cotinine levels, followed closely by adolescents, and adults had the lowest levels.

While all three measures of SHS exposure were lower in the fourth time period than in 1999–2000, they did not consistently decrease over time. In particular, there was an increase noted for 2003–2004 over the previous period for all measures and all age groups except children measured through self-reports. The multivariate regression of trend analyses indicates that the prevalence of self-reported exposure decreased significantly over time only for children. However, for adolescents and adults, both the prevalence of detectable cotinine and the geometric mean of cotinine levels proved to be statistically significantly decreasing over time. We further explored whether there might be trends for specific race/ethnic subgroups within an age group in a separate analysis. We found that none of the racial/ethnic subgroup for adolescents and adults exhibited any significant trend in self-reported exposure over time.

### 3.2. Self-Reported SHS Exposure

Table 2 shows the percentage of nonsmokers who report SHS exposure in the home during the combined years of 1999–2006 for the three age groups disaggregated by sociodemographic characteristics. The prevalence rates of SHS exposure were 20.8%, 18.7%, and 14.5% respectively for children, adolescent, and adults. Prevalence rates of exposure were significantly higher for female children and adults compared to males, but were similar for male and female adolescents. Non-Hispanic Blacks had the highest exposure rates of all race/ethnic groups. These rates were significantly different from the rates for non-Hispanic white children and adults, even after controlling for other sociodemographic factors; however, the odds ratio for black children is less than one and significant. Mexican American children and adolescents had the lowest prevalence rates of exposure and their lower rates were significantly different from the rates for their non-Hispanic white counterparts. Other Hispanic children and adolescents also had significantly lower exposure rates than non-Hispanic Whites. Children, adolescents, and adults born in Mexico had significantly lower exposure than those born in the U.S. Adolescents and adults born in foreign countries other than Mexico also had lower exposure than those who were U.S. born.

Education level was inversely related to self-reported SHS exposure for children and adolescents. Children and adolescents living in households headed by someone with less than a high school degree had exposure rates of 32.4% and 26.4% respectively. If the household was headed by someone with education beyond high school, these rates fell to a significantly lower level of 14.7% and 13.6. The exposure rates for adults with education beyond high school were significantly lower than for adults with less than a high school degree (11.9% compared to 17.4%).

Poverty status as measured by the PIR was also inversely related to self-reported SHS exposure. Children living in households above the poverty threshold (PIR ≥ 1.0) were significantly less likely to be exposed to SHS in the home than those below the poverty threshold. For children in high income (PIR ≥ 4.0) households, the difference was fivefold (35.3% vs 7.0%). For adolescents, the SHS exposure rates differed significantly by a factor of two for those below the poverty threshold compared to those in high income households (22.6% vs 10.9%). Adults showed the same pattern, though the differences were not statistically significant.

### 3.3. Cotinine Measured SHS Exposure

Table 3 shows the geometric mean of cotinine levels for the three age groups disaggregated by socioeconomic characteristics during the combined years of 1999–2006. Mean cotinine levels were 0.15 ng/mL for children, 0.11 ng/mL for adolescents, and 0.07 mg/mL for adults. Consistent with the self-reported SHS exposure results, adult females had significantly lower cotinine concentration than males. Black adolescents and adults had significantly higher mean cotinine levels than Whites, and Mexican Americans of all ages had significantly lower mean cotinine levels than their white counterparts. Nonsmokers of all ages born in Mexico and anywhere else outside the U.S. had significantly lower mean cotinine levels than those born in the U.S. Both educational level and poverty status were inversely related to the mean self-reported SHS exposure. Adults with education beyond high school, and children and adolescents living in a household with an educated head of household, had significantly lower mean cotinine levels than those with less than a high school education. Compared to those living below the poverty line, all other income groups had significantly lower mean cotinine levels for all three age groups except for low income (PIR = 1.0–1.99) adolescents.

The patterns of prevalence rates of detectable serum cotinine among nonsmokers by sociodemographic characteristics during 1999–2006 are similar to the results from Table 2 using self-reported SHS exposure, except that the absolute values of cotinine-measured prevalence rates are much higher. The prevalence rates of cotinine measured exposure were 59.0%, 52.4%, and 40.5% respectively for children, adolescent, and adults. The rates were particularly astonishingly high for non-Hispanic Blacks: 79.6% for children, 75.5% for adolescents, and 62.1% for adults. We do not show detailed results here, but instead only briefly point out some different patterns. Exposure rates differed by gender and country of birth only for adults, with females and those born in Mexico having significantly lower SHS exposure. Blacks of all ages had greater prevalence rates of detectable cotinine compared to Whites, while children of any other race/ethnicity groups and Mexican American adolescents were less likely to have detectable cotinine than Whites.

Non-Hispanic Blacks of all ages had significantly decreasing prevalence of detectable cotinine over the 8-year time period, and Mexican American children and adolescents had significantly decreasing trends in both the prevalence of cotinine and geometric mean of cotinine concentration over the same period.

### 3.4. Comparison of Self-Reported and Cotinine Measured SHS Exposure

Our results indicate much higher rates of cotinine measured exposure than self-reported SHS exposure. To further analyze how these exposure measures differ, we calculated the prevalence rates of detectable cotinine and the geometric means of cotinine concentration separately for nonsmokers who self-reported SHS exposure and those who did not (Table 4). 98.2% of children with self-reported exposure had detectable serum cotinine levels, with a geometric mean of 1.20 ng/mL. For children who did not have self-reported exposure, almost half (47.6%) of them nonetheless had detectable serum cotinine, with a mean value of 0.08 ng/mL. The pattern is similar for adolescents, though only 42% of those who report no home exposure had detectable cotinine, and the mean cotinine level for those with self-reported home exposure was lower than that of children: 0.75 ng/mL. For adults, the agreement between self-reported and cotinine-indicated exposure is greater for those who reported being exposed only in the home (94.2%) or both in the home and work (98.4%). For those who report being exposed only at work, 63.5% have detectable cotinine. More than one-third of adults (36.1%) who report that they were not exposed in the home or at work nonetheless had detectable cotinine levels. Geometric means of cotinine concentration were greatest for adults who report being exposed both in the home and at work (0.65 ng/mL), followed by those who report only home exposure (0.52 ng/mL). Levels were lowest for those who report being exposed only at work (0.11 ng/mL).

Multivariate logistic regression models were used to examine the relationship between self-reported SHS exposure and cotinine measured exposure. We regressed the presence of detectable cotinine (yes/no) on self-reported exposure (yes/no) adjusting for sociodemographic characteristics. For children and adolescents, only self-reported home exposure was included in the model. For adults, three self-reported variables, exposure at home only, at work only, and both at home and at work, were included in the model. The results show that self-reported SHS exposure is a statistically significant predictor of cotinine-indicated exposure with odds ratios of 43.5 and 46.0 for children and adolescents. For adults, the odds ratios were 90.0, 27.6 and 2.7 for exposure both in the home and at work, home only, and work only, respectively. Children and adolescents living in households headed by someone with education beyond high school, living in households above the poverty threshold, or who were Mexican American, were less likely to have detectable cotinine level after controlling for self-reported SHS exposure. Adults with education beyond high school or in the mid or high income group were less likely to have detectable cotinine level.

## 4. Discussion

Self-reported SHS exposure rates for children fell significantly between 1999 and 2006; however the prevalence rates of detectable cotinine and the mean cotinine levels did not show statistically significant trends. For adolescents and adults, the opposite occurred, with no significant trends in self-reported exposure, but a decrease in both the rate of people with detectable cotinine and the mean cotinine levels. All three SHS exposure measures are lower in the final time period compared to the first time period. However, the data for all measures show an increase during 2003–2004, and this might have contributed to a lack of statistically significant trends in exposure for some groups. Over the eight years we studied, serum cotinine concentration declined by 31% in children, 36% in adolescents, and 25% in adults. In contrast, Pirkle and colleagues [10] reported a 70% decline in serum cotinine concentration over the 14 years from 1988–2002. It is encouraging that the level of exposure continues to decline in more recent years.

Exposure patterns by demographic characteristics were similar when measured by either self-reports or serum cotinine. We found SHS exposure to be highest in children and also high in adolescents, consistent with earlier studies [10,11,13]. Among racial/ethnic groups, exposure rates and mean cotinine levels were highest in non-Hispanic Blacks across all age groups as documented in other studies [14–16], and lowest in Mexican American children [16] and adolescents. However, our findings for black children raise some interesting questions. While black children had higher rates of self-reported exposure, the adjusted odds ratio was less than one. This implies that after adjusting for the other demographic variables in the model, the exposure rates might have been lower. That would be consistent with previous findings indicating that blacks smoke fewer cigarettes per day, and lower intensity of smoking is associated with lower SHS exposure [12,24]. In fact, we observed that black children were more likely to be living in poor or low income households compared to white children (67% vs. 37%). Furthermore, black children’s exposure rates when stratified by poverty level were much lower than white counterparts for the poor group (36% vs. 51%) and low income group (25% vs. 34%) but were higher only for the high income group (12% vs. 7%). Thus, the inter-relationship among race/ethnicity, socioeconomic status, and SHS exposure merits further research. Our findings indicate that poor children have the highest exposure rates and level of exposure. While were encouraged to find that both self-reported and cotinine-indicated exposure rates showed a significant decline over the 8 years for poor children, the rates are still unacceptably high and there remains a need to focus more effort on reducing the health risk for this particularly vulnerable group.

We found that the prevalence rate of self-reported SHS exposure was much higher than the prevalence rate of cotinine measured exposure, as shown in other studies [9,24–26]. This could be explained by several factors. It is possible that people underreport their SHS exposure or that the survey designs lead to underreporting, particularly when self-reported SHS exposure is measured by the presence of a self-identified smoker in the household. It is also possible that the large proportion of the population with detectable cotinine is reflective of the widespread ubiquity of SHS in our environment and the challenges of controlling exposure in multiple settings. The self-reported exposure measure is based on questions pertaining to two environments only – the home and the workplace. However, serum cotinine is not limited to those settings, and might also reflect exposure in restaurants, other public and private places, and outdoors. There are also dietary sources of nicotine, and while these are unlikely to have a major impact on cotinine levels in the population as a whole, they may have an impact on some individuals. Our findings suggest that the lower self-reported exposure levels underestimate the extent of the continuing public health issue.

A number of limitations of this study need to be acknowledged. We did not have self-reported data on exposure for anyone in other indoor or outdoor settings. We did not account for the self-reported exposure in the workplace for adolescents. Data on workplace exposure for adults for 2005–2006 were not available at the time of our analyses. Serum cotinine is a good measure of SHS exposure during the previous 3–4 days. However, it has a short half-life and thus would not measure exposure that had occurred more than a few days ago. However, cotinine is still considered the biomarker of choice for SHS exposure assessment [1].

Despite many years of tobacco control efforts aimed at reducing SHS exposure in the home and workplace, exposure to SHS in the home and in the workplace continues to be an important public health problem. While the prevalence of exposure as well as the mean cotinine concentration are lower than they were decades earlier, people continue to be exposed. Children, adolescents, non-Hispanic Blacks, low income people, and people living in households headed by someone with low education remain particularly vulnerable.

## Figures and Tables

**Table 1.** Self-reported secondhand smoke exposure and cotinine measured exposure among nonsmokers by year and age group^1^: U.S., 1999–2006.

	**1999–2000**		**2001–2002**		**2003–2004**		**2005–2006**		**1999–2006**

	**Sample Size^2^**	**Mean (95% CI)**	**Sample Size^2^**	**Mean (95% CI)**	**Sample Size^2^**	**Mean (95% CI)**	**Sample Size^2^**	**Mean (95% CI)**	**Time Trend**
**Children (3–11)**									
Self-Reported Home Exposure (%) 3	1556	22.7 (18.5–26.8)	1789	23.7 (19.1–28.3)	1550	22.3 (15.4–29.2)	1759	14.6 (10.4–18.8)	*
Cotinine Measured Exposure (%) 4	1179	64.5 (56.4–72.6)	1423	56.0 (47.8–64.1)	1265	65.2 (56.2–74.3)	1300	50.9 (45.8–56.0)	
Geometric Mean Cotinine (ng/mL)	1179	0.16 (0.12–0.20)	1423	0.16 (0.12–0.20)	1265	0.18 (0.12–0.26)	1300	0.11 (0.09–0.13)	
**Adolescents (12–19)**									
Self-Reported Home Exposure (%) 3	1517	23.4 (19.4–27.3)	1610	16.0 (12.5–19.6)	1524	21.5 (16.1–27.0)	1519	15.2 (10.9–19.6)	
Cotinine Measured Exposure (%) 4	1530	63.2 (56.6–69.9)	1623	45.7 (35.4–56.0)	1536	56.6 (50.1–63.2)	1533	47.0 (40.2–53.8)	*
Geometric Mean Cotinine (ng/mL)	1530	0.14 (0.12–0.16)	1623	0.10 (0.08–0.12)	1536	0.13 (0.11–0.16)	1533	0.09 (0.08–0.11)	*
**Adults (20+)**									
Self-Reported Home 3 or Work Exposure (%)	2885	15.7 (13.0–18.3)	3317	13.1 (12.1–14.2)	3118	14.8 (12.9–16.6)	3185	NA	
Home only (%)	2885	5.8 (4.8–6.9)	3317	5.3 (4.6–6.0)	3118	5.4 (4.1–6.7)	3185	NA	
Work only (%)	2885	8.7 (6.4–11.1)	3317	7.2 (6.4–8.1)	3118	8.5 (7.6–9.4)	3185	NA	
Home and Work (%)	2885	1.1 (0.5–1.6)	3317	0.7 (0.2–1.1)	3118	0.8 (0.2–1.5)	3185	NA	
Cotinine Measured Exposure (%) 4	2919	47.4 (42.3–52.5)	3348	37.3 (31.6–43.1)	3139	42.4 (35.3–49.5)	3206	35.8 (32.6–39.0)	*
Geometric Mean Cotinine (ng/mL)	2919	0.08 (0.07–0.09)	3348	0.07 (0.06–0.08)	3139	0.07 (0.06–0.08)	3206	0.06 (0.06–0.07)	*

^1^ Data source: National Health and Nutrition Examination Survey.

^2^ Unweighted

^3^ The presence of at least one household member who smokes in the home.

^4^ Detectable serum cotinine ≥ 0.05 ng/mL.

* Significance of time trend analysis, P-value < 0.05.

NA: Exposure rates are not reported because work exposure data are not yet available.

Note: All the estimates are based on weighted analyses accounting for complex survey design.

Adult self-reported exposures may not sum to total due to rounding.

**Table 2.** Percentages of nonsmokers with self-reported secondhand smoke exposure^1^ by socio-demographic characteristics and age group, and odds ratios from multivariate logistic regression model^1^: U.S., 1999–2006.

	**Children (3–11)**			**Adolescents (12–19)**			**Adults (20+)^2^**		

**Characteristic**	**Sample Size^3^**	**Unadjusted %**	**Adjusted Odds Ratio (95% CI)**	**Sample Size^3^**	**Unadjusted %**	**Adjusted Odds Ratio (95% CI)**	**Sample Size^3^**	**Unadjusted %**	**Adjusted Odds Ratio (95% CI)**

**Total**	6654	20.8		6170	18.7		9320	14.5	
**Gender**									
Male (reference)	3307	19.6		2967	18.2		3939	18.7	
Female	3347	22.2	1.24 (1.06–1.46)*	3203	19.2	1.04 (0.85–1.26)	5381	11.3	0.52 (0.45–0.61)*
**Race/Ethnicity**									
Non-Hispanic White (reference)	1834	23.1		1494	20.1		4775	13.3	
Non-Hispanic Black	2143	27.0	0.64 (0.50–0.81)*	2033	25.4	0.87 (0.67–1.13)	1567	19.9	1.59 (1.30–1.93)*
Mexican American	2120	8.5	0.14 (0.10–0.21)*	2240	9.4	0.24 (0.17–0.36)*	2282	15.7	1.22 (0.95–1.58)
Other Hispanic	293	17.3	0.40 (0.24–0.65)*	230	10.6	0.36 (0.18–0.73)*	283	18.4	1.36 (0.96–1.91)
Other Race (including multi-racial)	264	14.3	0.46 (0.26–0.81)*	173	13.7	0.53 (0.27–1.02)	413	15.9	1.85 (1.25–2.74)*
**Country of Birth**									
US (reference)	6213	21.4		5245	19.7		7005	14.5	
Mexico	287	5.3	0.37 (0.21–0.66)*	658	6.1	0.40 (0.25–0.64)*	1336	14.6	0.69 (0.52–0.92)*
Elsewhere	153	14.1	0.55 (0.28–1.06)	266	10.6	0.49 (0.29–0.84)*	976	14.3	0.74 (0.56–0.99)*
Unknown	1	–	–	1	–	–	3	–	–
**Education Level 4**									
< High School (reference)	1999	32.4		2006	26.4		2840	17.4	
High School or GED	1492	26.7	0.66 (0.50–0.86)*	1332	26.0	0.70 (0.48–1.03)	2043	18.7	1.15 (0.90–1.48)
> High School	3050	14.7	0.41 (0.29–0.59)*	2687	13.6	0.34 (0.24–0.47)*	4421	11.9	0.70 (0.55–0.89)*
Unknown	113	28.8	1.03 (0.47–2.25)	145	29.1	1.10 (0.54–2.24)	16	–	–
**Poverty Income Ratio**									
0 – 0.99 (reference)	2151	35.3		1795	22.6		1344	16.8	
1 – 1.99	1736	25.2	0.56 (0.41–0.77)*	1512	24.4	1.16 (0.82–1.63)	2232	16.4	0.98 (0.76–1.27)
2 – 3.99	1498	16.1	0.33 (0.23–0.48)*	1532	18.9	0.83 (0.60–1.15)	2425	15.6	0.96 (0.75–1.22)
≥ 4.0	876	7.0	0.13 (0.08–0.21)*	955	10.9	0.48 (0.28–0.81)*	2617	12.1	0.78 (0.58–1.05)
Unknown	393	16.6	0.35 (0.23–0.54)*	376	18.9	0.83 (0.55–1.25)	702	14.4	0.89 (0.64–1.25)

^1^ Data source: National Health and Nutrition Examination Survey.

^2^ For adults, exposure rates are analyzed by considering both work exposure and home exposure based on the NHANES data in 1999–2004.

^3^ Unweighted

^4^ For children and adolescents, these numbers reflect the household reference person's status.

* Odds ratio is statistically significant at P-value < 0.05.

–Results are not shown for sample sizes of 20 or smaller.

Note: All the exposure rates and odds ratios are based on weighted analyses accounting for complex survey design.

**Table 3.** Geometric mean cotinine levels among nonsmokers by sociodemographic characteristics and age group, and multivariate regression coefficients^1^: U.S., 1999–2006.

	**Children (3–11)**			**Adolescents (12–19)**			**Adults (20+)**		

**Characteristic**	**Sample Size**	**Unadjusted Geometric Mean Cotinine (ng/mL)**	**Regression Coefficient**	**Sample Size^2^**	**Unadjusted Geometric Mean Cotinine (ng/mL))**	**Regression Coefficient**	**Sample Size^2^**	**Unadjusted Geometric Mean Cotinine (ng/mL)**	**Regression Coefficient**

**Total**	**5167**	**0.15**		**6222**	**0.11**		**12612**	**0.07**	
**Gender**									
Male (reference)	2606	0.14		2997	0.11		5348	0.08	
Female	2561	0.15	0.073	3225	0.11	−0.072	7264	0.06	−0.251*
**Race/Ethnicity**									
Non-Hispanic White (reference)	1395	0.16		1505	0.11		6399	0.07	
Non-Hispanic Black	1648	0.28	−0.103	2045	0.20	0.283*	2253	0.12	0.486*
Mexican American	1705	0.07	−1.461*	2263	0.07	−0.857*	3023	0.06	−0.124*
Other Hispanic	228	0.10	−0.893*	234	0.09	−0.372*	531	0.07	0.019
Other Race (including multi-racial)	191	0.13	−0.273	175	0.09	−0.301*	406	0.07	0.167
**Country of Birth**									
U.S. (reference)	4821	0.15		5290	0.11		9480	0.07	
Mexico	228	0.07	−0.571*	662	0.07	−0.310*	1818	0.06	−0.317*
Elsewhere	118	0.09	−0.669*	269	0.09	−0.293*	1311	0.07	−0.200*
Unknown	0	–	–	1	–	–	3	–	–
**Education Level^2^**									
< High School (reference)	1607	0.28		2014	0.17		3704	0.09	
High School or GED	1178	0.24	−0.202*	1341	0.17	−0.091	2766	0.08	−0.038
> High School	2285	0.10	−0.788*	2696	0.08	−0.632*	6126	0.06	−0.279*
Unknown	97	0.25	0.034	171	0.15	−0.145	16	–	–
**Poverty Income Ratio**									
0 – 0.99 (reference)	1725	0.34	−0.529*	1795	0.18	−0.118	1814	0.10	−0.226*
1 – 1.99	1361	0.21	−1.143*	1512	0.15	−0.458*	2982	0.08	−0.380*
2 – 3.99	1126	0.10	−1.692*	1532	0.10	−0.820*	3368	0.07	−0.503*
≥ 4.0	637	0.06	−0.684*	955	0.07	−0.327*	3529	0.06	−0.219*
Unknown	318	0.17		428	0.12		919	0.08	

^1^ Data source: National Health and Nutrition Examination Survey.

^2^ Unweighted

^2^ For children and adolescents, these numbers reflect their household reference person's status.

– Results are not shown for sample size of 20 or smaller.

* Regression coefficient is statistically significant at P-value < 0.05.

Note: All the cotinine means and regression coefficients are based on weighted analyses accounting for complex survey design.

**Table 4.** Comparison of self-reported secondhand smoke exposure and cotinine measured exposure for nonsmokers by age group^1^: U.S., 1999–2006.

	**Sample Size (unweighted)**	**% with Detectable Cotinine Level ≥ 0.05 ng/mL (95% CI)**	**Geometric Mean Cotinine, ng/mL**
**Children (3–11)**			
Self-Reported Home Exposure	1060	98.2 (97.2–99.3)	1.20
No Self-Reported Home Exposure	4053	47.6 (43.6–51.6)	0.08
**Adolescents (12–19)**			
Self-Reported Home Exposure	1101	97.4 (96.4–98.4)	0.75
No Self-Reported Home Exposure	5069	42.0 (37.7–46.2)	0.07
**Adults (20+)^2^**			
Self-Reported Home or Work Exposure	1237	77.2 (73.1–81.3)	0.22
Self-Reported Home Exposure Only	527	94.2 (91.9–96.5)	0.52
Self-Reported Work Exposure Only	640	63.5 (57.1–69.8)	0.11
Self-Reported Home and Work Exposure	70	98.4 (95.9–100.0)	0.65
No Self-Reported Home or Work Exposure	8083	36.1 (32.7–39.5)	0.06

^1^ Data source: National Health and Nutrition Examination Survey.

^2^ For adults, exposure rates are analyzed and compared based on the NHANES data in 1999–2004.

Note: All the estimates are based on weighted analyses accounting for complex survey design.

## References

[1] US Department of Health and Human Services The health consequences of involuntary exposure to tobacco smoke: A report of the Surgeon General US DHHS, Centers for Disease Control and Prevention, Office of Smoking and Health Washington, D.C., U.S. 2006 20669524

[2] California Environmental Protection Agency Proposed Identification of Environmental Tobacco Smoke as a Toxic Air Contaminant California Environmental Protection Agency, Office of Environmental Health Hazard Assessment Sacramento, CA, U.S. 2005

[3] Hackshaw A Law M Wald N The accumulated evidence on lung cancer and environmental tobacco smoke Br. Med. J 1997 315 980 999 9365295 10.1136/bmj.315.7114.980 PMC2127653

[4] Jinot J Bayard S Respiratory health effects of exposure to environmental tobacco smoke Rev. Environ. Health 1996 11 89 100 9000301 10.1515/reveh.1996.11.3.89

[5] Mannino DM Moorman JE Kingsley B Rose D Repace J Health effects related to environmental tobacco smoke exposure in children in the United States: data from the Third National Health and Nutrition Examination Survey Arch. Pediatr. Adolesc. Med 2001 155 36 41 11177060 10.1001/archpedi.155.1.36

[6] Barnoya J Glantz SA Cardiovascular effects of secondhand smoke: nearly as large as smoking Circulation 2005 111 2684 2698 15911719 10.1161/CIRCULATIONAHA.104.492215

[7] Thun M Henley J Apicella L Epidemiologic studies of fatal and nontatal cardiovascular disease and secondhand smoke exposure from spousal smoking Environ. Health Perspect 1999 107 841 846 10592140 10.1289/ehp.99107s6841 PMC1566204

[8] Steenland K Risk assessment for heart disease and workplace secondhand smoke exposure among nonsmokers Environ. Health Perspect 1999 107 859 863 10592143 10.1289/ehp.99107s6859 PMC1566202

[9] Pirkle JL Flegal KM Bernert JT Brody DJ Etzel RA Maurer KR Exposure of the US population to environmental tobacco smoke: the Third National Health and Nutrition Examination Survey, 1988 to 1991 JAMA 1996 275 1233 1240 8601954

[10] Pirkle JL Bernert JT Caudill SP Sosnoff CS Pechacek TF Trends in the exposure of nonsmokers in the U.S. population to secondhand smoke: 1988–2002 Environ. Health Perspect 2006 114 853 858 16759984 10.1289/ehp.8850 PMC1480505

[11] Schober SE Zhang C Brody DJ Disparities in secondhand smoke exposure – United States, 1988–1994 and 1999–2004 MMWR Morb. Mortal. Wkly. Rep 2008 57 744 747 18614993

[12] Soliman S Pollack HA Warner KE Decrease in the prevalence of environmental tobacco smoke exposure in the home during the 1990s in families with children Am. J. Public Health 2004 94 314 320 14759948 10.2105/ajph.94.2.314 PMC1448249

[13] Machlin SR Hill SC Liang L Children living with adult smokers, United States, 2004 Statistical Brief # 147 Agency for Healthcare Research and Quality Rockville, MD 2006Available online: http://www.meps.ahrq.gov/mepsweb/data_files/publications/st147/stat147.pdf (accessed February 2009)

[14] Wagenknecht LE Manolio TA Sidney S Burke GL Haley NJ Environmental tobacco smoke exposure as determined by cotinine in black and white young adults: the CARDIA study Environ. Res 1993 63 39 46 8404773 10.1006/enrs.1993.1124

[15] Scarcinci IC Watson JM Slawson DL Klesges RC Murray DM Eck-Clemens LH Socioeconomic status, ethnicity, and environmental tobacco exposure among non-smoking females Nicotine Tob. Res 2000 2 355 361 11197316 10.1080/713688150

[16] Sexton K Adgate JL Church TR Hecht SS Ramachandran G Greaves IA Fredrickson AL Ryan AD Carmella SG Geisser MS Children’s exposure to environmental tobacco smoke: Using diverse exposure metrics to document ethnic/racial differences Environ. Health Perspect 2004 112 392 397 14998759 10.1289/ehp.6473 PMC1241873

[17] Pletsch PK Environmental tobacco smoke exposure among Hispanic women of reproductive age Public Health Nurs 1994 11 229 235 7937494 10.1111/j.1525-1446.1994.tb00416.x

[18] National Center for Health Statistics (NCHS) National Health and Nutrition Examination Survey Response Rates, 1999–2006 Department of Health and Human Services, Centers for Disease Control and Prevention Hyattsville, MD, U.S. Available online: http://www.cdc.gov/nchs/about/major/nhanes/nhanes_cps_totals.htm (accessed January 15, 2009).

[19] U.S. Census Bureau How the Census Bureau Measures PovertyAvailable online: http://www.census.gov/hhes/www/poverty/povdef.html 2003(accessed May 13, 2009).

[20] Office of Management and Budget Statistical Policy Directive No 14 Definition of poverty for Statistical Purposes 5 1978Available online: http://www.census.gov/hhes/www/poverty/povmeas/ombdir14.html (accessed May 13, 2009).

[21] SAS Institute Inc SAS 9.1.3 Help and Documentation SAS Institute Inc.Cary, NC, U.S. 2000 2004

[22] Mannino DM Caraballo R Neal Benowitz N Repace J Predictors of Cotinine Levels in US Children Chest 2001 120 718 724 11555498 10.1378/chest.120.3.718

[23] Ellis JA Gwynn C Garg RK Philburn R Aldous KM Perl SB Thorpe L Frieden TR Secondhand smoke exposure among nonsmokers nationally and in New York City Nicotine Tob Res 2009doi:10.1093/ntr/ntp02. 10.1093/ntr/ntp021 19351780

[24] Hassmiler KM Warner KE Mendez D Levy DT Romano E Nondaily smokers: who are they? Am. J. Public Health 2003 93 1321 1327 12893622 10.2105/ajph.93.8.1321 PMC1447964

[25] Campuzano JC Hernandez-Avila M Jaakkola MS Lazcano-Ponce E Kuri-Morales P Bautista P Benowitz NZ Ceraso M Blackford A Samet JM Determinants of salivary cotinine levels among current smokers in Mexico Nicotine Tob. Res 2004 6 997 1008 15801572 10.1080/14622200412331324956

[26] Emmons KM Abrams DB Marshall R Marcus BH Kane M Novotny TE Etzel RA An evaluation of the relationship between self-report and biochemical measures of environmental tobacco smoke exposure Prev. Med 1994 23 35 39 8016030 10.1006/pmed.1994.1005

[27] Martinez-Sanchez JM Fernandez E Fu M Pascual JA Ariza C Agudo A Borràs JM Schiaffino A Moncada A Jané M Saltó E Assessment of exposure to secondhand smoke by questionnaire and salivary cotinine in the general population of Barcelona, Spain (2004–2005) Prev Med 2009In press. 10.1016/j.ypmed.2008.12.020 19166873

